# Abiotic predictors and annual seasonal dynamics of *Ixodes ricinus*, the major disease vector of Central Europe

**DOI:** 10.1186/s13071-015-1092-y

**Published:** 2015-09-18

**Authors:** Milan Daniel, Marek Malý, Vlasta Danielová, Bohumír Kříž, Patricia Nuttall

**Affiliations:** National Institute of Public Health, Prague, Czech Republic; Department of Zoology, University of Oxford, South Parks Road, Oxford, OX1 3PS UK; NERC Centre for Ecology & Hydrology, Wallingford, Oxfordshire UK

**Keywords:** Tick, *Ixodes ricinus*, Co-occurrence, Temperature, Daylight length, Extreme weather event

## Abstract

**Background:**

Abiotic conditions provide cues that drive tick questing activity. Defining these cues is critical in predicting biting risk, and in forecasting climate change impacts on tick populations. This is particularly important for *Ixodes ricinus* nymphs, the vector of numerous pathogens affecting humans.

**Methods:**

A 6-year study of the questing activity of *I. ricinus* was conducted in Central Bohemia, Czech Republic, from 2001 to 2006. Tick numbers were determined by weekly flagging the vegetation in a defined 600 m^2^ field site. After capture, ticks were released back to where they were found. Concurrent temperature data and relative humidity were collected in the microhabitat and at a nearby meteorological station. Data were analysed by regression methods.

**Results:**

During 208 monitoring visits, a total of 21,623 ticks were recorded. Larvae, nymphs, and adults showed typical bimodal questing activity curves with major spring peaks and minor late summer or autumn peaks (mid-summer for males). Questing activity of nymphs and adults began with ~12 h of daylight and ceased at ~9 h daylight, at limiting temperatures close to freezing (in early spring and late autumn); questing occurred during ~70 % calendar year without cessation in summer. The co-occurrence of larvae and nymphs varied annually, ranging from 31 to 80 % of monitoring visits, and depended on the questing activity of larvae. Near-ground temperature, day length, and relative air humidity were all significant predictors of nymphal activity. For 70 % of records, near-ground temperatures measured in the microhabitat were 4–5 °C lower than those recorded by the nearby meteorological observatory, although they were strongly dependent. Inter-annual differences in seasonal numbers of nymphs reflected extreme weather events.

**Conclusions:**

Weather predictions (particularly for temperature) combined with daylight length, are good predictors of the initiation and cessation of *I. ricinus* nymph questing activity, and hence of the risk period to humans, in Central Europe. Co-occurrence data for larvae and nymphs support the notion of intrastadial rather than interstadial co-feeding pathogen transmission. Annual questing tick numbers recover quickly from the impact of extreme weather events.

**Electronic supplementary material:**

The online version of this article (doi:10.1186/s13071-015-1092-y) contains supplementary material, which is available to authorized users.

## Background

The ixodid tick species, *Ixodes ricinus*, is the most important vector of pathogens in Europe, transmitting the aetiological agents of debilitating diseases such as Lyme borreliosis and tick-borne encephalitis in humans, louping ill in sheep, and babesiosis in cattle and dogs. Generally, infections are acquired by larvae feeding on infected vertebrate hosts (often rodents) and transmitted by nymphs, while successful feeding of adult females determines the size of the tick population and hence the host contact rate. Vertical transmission rates of tick-borne pathogens are usually <1 % [1, 2]. *Ixodes ricinus* is a generalist, 3-host tick, feeding on different individual vertebrate hosts at the larval, nymphal and adult stages. The periods of host parasitism amount to <2 % of the duration of the tick’s life cycle (typically 2–3 years) during which the tick remains attached and feeding on its host for 2–10 days, unaffected by abiotic conditions (unless feeding on poikilothermic hosts). By contrast, nearly all the tick’s life cycle is spent in the surface layers of soil or forest litter where environmental conditions influence development [3, 4]. Development is synchronized with seasonal climatic conditions by morphogenetic diapause, during which metamorphosis of engorged larvae and nymphs is delayed, and behavioural diapause manifested as cessation of questing activity [5]. Nonparasitic phases require a microclimatic relative humidity of ≥80 % to avoid fatal desiccation, and little or no development takes place <10 °C [3, 6, 7]. Given the importance of *I. ricinus* as a disease vector, the yearly time of initiation and duration of questing are critical parameters in managing the risk of being bitten, particularly for nymphs as they are the most frequent stage attacking humans and thus the most important transmitter of tick-borne infections [8–12]. However, despite numerous studies of the effect of environmental conditions on tick activity, there have been few long-term systematic studies of micro- and macro-abiotic conditions (temperature, relative humidity, and daylight length) and questing obtained by field investigations, the critical importance of which has been emphasized [13].

Here we present the results of a 6-year field study of questing activity of *I. ricinus* in Central Bohemia. The research began in 2001 within the framework of the WHO/EC project, Climate Change and Adaptation Strategies for Human Health. The period included 2002 and 2003 when exceptional meteorological situations during summer caused flooding and a severe heat wave in the Czech Republic and across Central Europe [14]. The primary aim was to determine how well routine weather forecasts could be used to predict the risk to humans of being bitten by *I. ricinus*. The study site is in an area where *I. ricinus* is infected with tick-borne encephalitis virus, *Borrelia burgdorferi* sensu stricto, *B. afzelii*, and *B. garinii*, and cases of human Lyme borreliosis (caused by *B. afzelii*) have been reported. During the 6-year field investigation of questing tick activity, temperature and relative humidity were measured directly in the selected monitoring site and concurrent macroclimatic changes registered at the local meteorological observatory (Czech Hydrometeorological Institute, CHMI). These conditions were selected after an initial study (2001–2002) of the influence of weather conditions (daily sunshine; average, maximum and minimum air temperature; wind speed, soil temperature, precipitation, air humidity, and soil moisture) showed that tick behavior could be predicted by two models, one dependent on soil moisture and the other on air temperature [15].

The concept and methodology of the study were based on previous studies of *I. ricinus* field ecology in natural foci of TBE virus in the Czech Republic and southern Europe (Bulgaria and former Yugoslavia) [16]. Conditions were strictly defined for the monitoring site, time course, and meterological observations (see Methods). These conditions match those recommended by others to reduce sampling error and bias in field studies of *I. ricinus* questing activity [4, 13, 17–20]. The protocol adopted enabled us to make a statistically robust comparison of tick questing activity and abiotic conditions, and hence to determine if weather conditions can be used to predict risk to humans of acquiring tick-borne infections.

## Methods

### Study area

**Fig. 1:**
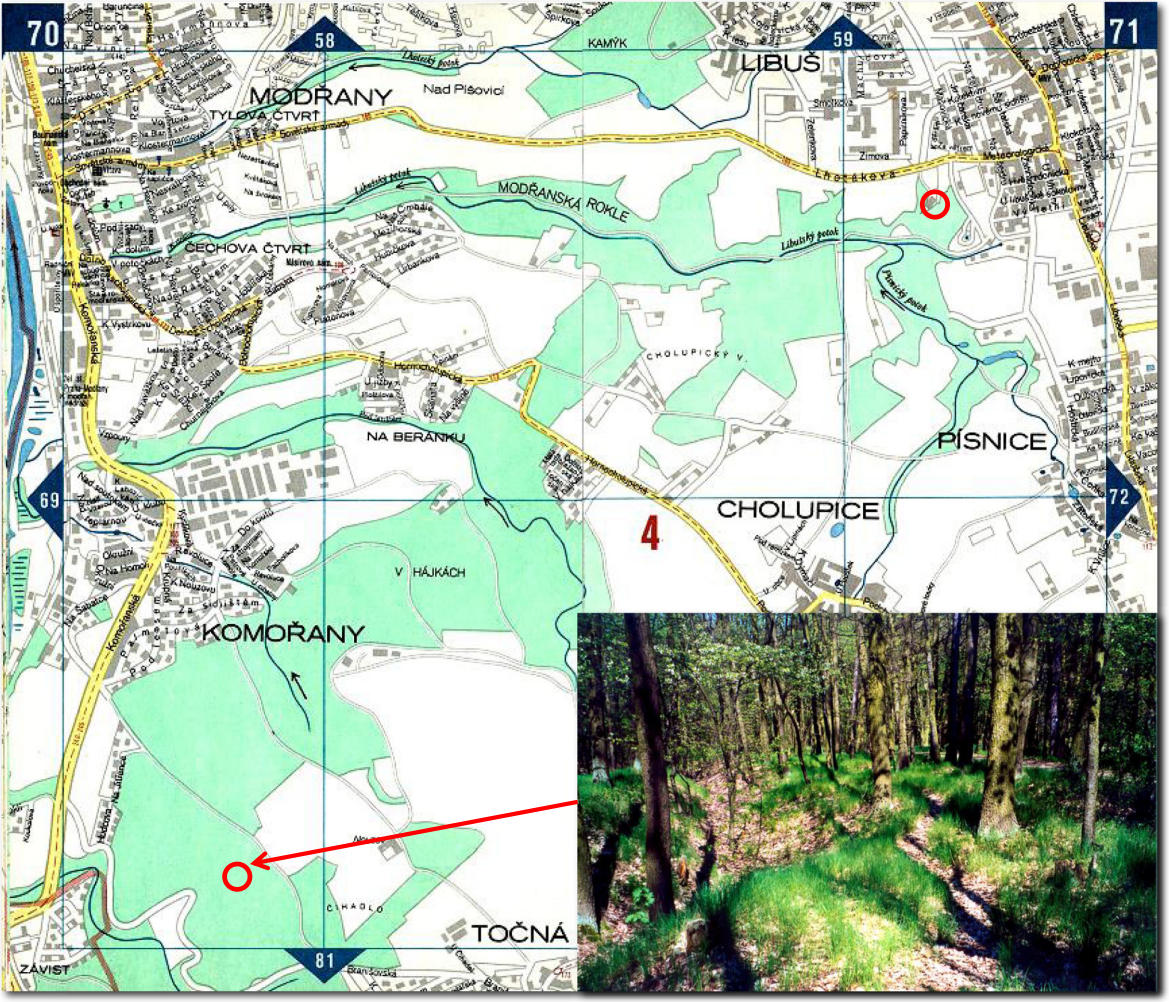
Location and habitat of the monitoring site. Circle indicates location of the Czech Hydrometeorological Institute in Prague-Libuš; circle and arrow indicate location of the monitoring site. Insert shows typical habitat of the monitoring site

The selected area for tick monitoring is located in the southern outskirts of Prague (49°58′43″N; 14°24′52″E; altitude 325 MSL), 4 km from the main observatory station of the Czech Hydrometeorological Institute (CHMI) in Prague-Libuš, one of the best equipped observatories in the Czech Republic (Fig. 1). An adequate size (eg. 600 m^2^) was required to provide a large enough sample size for registering the spring start and autumn cessation of questing activity when numbers of active ticks are comparatively low. The site comprises eutrophicated, acid oak wood (*Quercus robur*, 70–100 year-old trees), with patches of younger hornbeam (*Carpinus betulus*). Most of the stand is evenly dense, with a few small openings. The tree canopy cover is 70 % and the shrub canopy cover (*Rubus fruticosa*, *Sambucus nigra*) is <10 %. The herb canopy cover is ~25 %, with prevailing low (<20 cm) sparse grass (mostly *Poanemoralis*) and patches completely covered with dead leaves or moss. In this vegetation community, considered typical habitat for *I. ricinus* in Central Europe, three adjacent plots were established each of 200 m^2^ having all the features described, which were consistent year-on-year. Low vegetation of the undergrowth (remaining <20 cm in height throughout the year) in the monitored plots limited the possible bias from seasonal growth of the vegetation on the monitoring of ticks and microclimate. The habitat under study is situated on flat ground completely exposed to sunlight giving homogenous conditions over the whole site. Although the study area is under forestry management, with a controlled population of game (including roe deer), there was no forestry activity on the site or in the surrounding area; the absence of fencing allows free movement of all animals (including humans).

### Tick monitoring

A 6 year monitoring period was chosen based on the assumption this allowed completion of three generations of *I. ricinus* [3]. Questing activity of *I. ricinus* was investigated by the standard flagging method [21] in the defined plots, at weekly intervals, between 09.00 and 12.00 h, from March to November, 2001–2006. One week frequency at defined intervals ensured sufficient granularity and reproducibility of measurements while being logistically feasible. On 27 occasions, flagging was delayed by one day due to rain, and the meteorological data matched accordingly. Spring investigations began when environmental conditions in the study area became suitable for flagging (no consistent snow or ice coverings). Autumn investigations ended when two consecutive flagging samples were negative. The period between late morning and noon was selected as the most appropriate based on previous studies of diurnal variations of *I. ricinus* activity and ignoring night time questing because the epidemiological risk is low (our own unpublished data; [9]). The flag (50 × 70 cm) was white flannel fabric with a slight nap. All plants and patches covered with dead leaves or moss were flagged. The flag was checked on both sides after 2 m were flagged. In total, 208 monitoring visits were completed. Larvae, nymphs, females, and males were identified and counted separately. Ticks were not removed from the plots but were immediately released back to where they were collected.

### Temperature and humidity measurements

Near-ground air temperatures were measured using a mercury thermometer graduated in 0.1 °C increments placed at ~1 cm above the ground (but not touching the ground), in shadow, away from the sun. Previous studies showed that air temperature measured at heights ranging up to 30 cm above ground in the study site did not differ significantly under different synoptic weather conditions [19]. Temperatures were read at 09.00, 10.00 and 11.00 h during each monitoring visit (2001–2006). For all records, near-ground temperature within one monitoring visit varied by ≤5 °C. Near-ground air humidity (RH %) was measured during 2005 and 2006 at the same time points using an electronic hygrometer (Hygrocheck Relative Humidity Tester, Hanna Instruments, Portugal). The instrument was regularly checked for accuracy against an Assman aspirated hygrometer under laboratory conditions. In analyses relating to tick questing activity, microclimatic data collected directly on the monitoring plots were used, unless stated otherwise. Additional meteorological data were obtained from the database of CHMI. The numbers of daylight hours were calculated for the following geographic coordinates: 50°00´N, 15°00´E.

The monitoring period was divided into seasonal periods according to the course of the normal daily temperature, i.e. the 30-year average of daily mean air temperature in the Czech Republic. These periods are defined as:

- “spring” = normal daily temperature <10 °C; from commencement of monitoring until 29 April inclusive.
- “summer” = normal daily temperature >10 °C; from 30 April to 8 October.
- “autumn” = normal daily temperature <10 °C; from 9 October to the end of the monitoring period.

The defining temperature of 10 °C was based on observations that development of *I. ricinus* occurs at and above this threshold [6, 7].

### Statistical analyses

Locally weighted scatter plot smoothing (LOWESS) was used for regression analysis of numbers of active ticks against time. For comparison of the patterns of curves between stages, the data were first standardised by subtracting the mean from the observed number of ticks and dividing by the resulting standard deviation. A generalized additive linear model with Poisson distribution and log link was used to model the effect of year and smoothed effect of seasonality. The model was fitted simultaneously by penalized spline. Parametric bootstrap with 1000 replicates was used for construction of bias-corrected 95 % confidence intervals for week of maximum tick activity. For comparison between categories, the Kruskal-Wallis test was used, followed by the multiple comparisons procedure. To model the occurrence of *I. ricinus* depending on possible predictors, Poisson regression was used. The degree of association between temperature measurements for two sources was quantified by the Spearman correlation coefficient, *r*_*s*_. All tests were evaluated at a significance level of 0.05. The data were analyzed using the Stata software package, release 9.2 (Stata Corporation, College Station, U.S.A.).

## Results

### Comparative seasonal dynamics of larvae, nymphs and adults

**Fig. 2:**
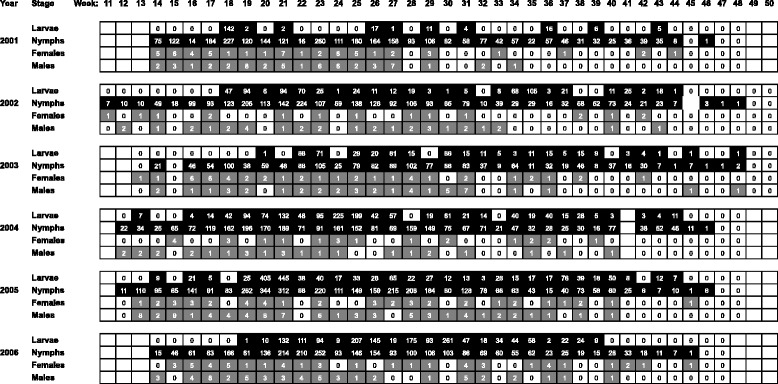
Numbers and co-occurrence of larvae, nymphs, and adults recorded during the six year monitoring period. Each monitoring day within a calendar week is depicted by an open cell, which is shaded to indicate presence of ≥1 larva, nymph, adult female or adult male. Immature stages are shaded black and adults are shaded grey. There was no monitoring visit in week 41 of 2004

**Fig. 3:**
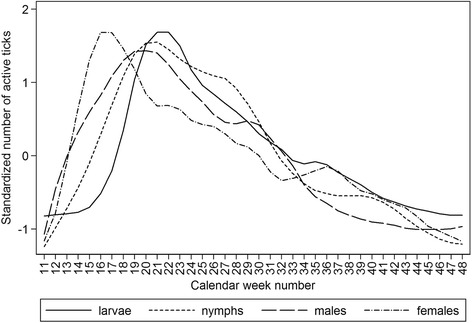
Standardized mean numbers of questing ticks during the monitoring period 2001-2006, smoothed by locally weighted regression. The prevalence data by stage are standardized by conversion to the same scale to be comparable (see Methods)

Over the 6-year monitoring period, a total of 21,623 ticks were recorded comprising 5690 larvae, 15,405 nymphs, 231 females, and 297 males (Fig. 2). Each stage showed a typical bimodal seasonal pattern of activity, with peak activity reached first by females (week 17, 95 % CI 11.6–19.6), and then males (week 20, 95 % CI 16.1–23.1), nymphs (week 21, 95 % CI 21.0–21.0) and larvae (week 22, 95 % CI22.0–22.0, at the end of spring) (Fig. 3). Overall, there were significant differences between the stages (*p* < 0.001); female activity was significantly different from that of larvae and nymphs, but was not different from males. Peak activity was followed by a declining shoulder of questing numbers with a much less pronounced peak in late summer–autumn although the second peak of male tick activity occurred in mid-summer (July); there was no detectable autumn peak of male activity. Nymphs or nymphs and adults were found at the first opportunity for monitoring to commence, the earliest being week 11 (early March) although questing ticks were never found during *ad hoc* visits in January (Fig. 2). Nymphs or nymphs and adults were last found in week 48 (late November) although more usually in late October. Larvae were first collected in mean week 17 and last collected at mean week 44. The mean periods of activity were: nymphs (34.7 ± 1.9 weeks), females (30.3 ± 2.8 weeks), males (28.6 ± 4.8 weeks), and larvae (27.7 ± 3.6 weeks). Nymphs were collected on almost all monitoring visits during their activity period (mean 98.1 ± 1.5 %), with fewer collections of larvae (79.7 ± 21.7 %), males (70.4 ± 11.0 %) and females (60.3 ± 12.3 %) (Fig. 2).

Analysis of the monitoring dataset for presence/absence of each developmental stage revealed the co-occurrence of all questing stages of *I. ricinus* ticks during 55 monitoring visits (i.e. 26.3 ± 11.5 % annual monitoring visits) at varying intensity levels (Fig. 2). The co-occurrence of larvae and nymphs varied markedly: from 31 % in 2001 to 80 % in 2005, with a median incidence of 65 %. There was no occasion when larvae were present and nymphs were apparently absent, whereas, during 7.0 ± 3.4 weeks of the tick activity period, nymphs were collected but larvae were not. Concurrent collection of questing females and males ranged from 28 % in 2002 to 71 % in 2005, with a median incidence of 62 %.

### Comparison of questing activity and ecoclimatic conditions

During the 6 year monitoring period, questing nymphs and adults were first recorded between weeks 11 and 14 under the following conditions: 11.6–13.2 h of daylight, microclimatic temperature range −3 °C to 11 °C, and CHMI temperature range 2.3 °C to 12.8 °C. In 2006, exceptional spring meteorological conditions were recorded, with the monitoring plots remaining snow covered until week 13; similarly freezing conditions (mean daily temperature ≤2 °C) occurred in 2001 but without snow. Climatic conditions consistent with successful completion of the life cycle (temperatures >10 °C) were recorded for weeks 18 to 40 (i.e. a span of 23 weeks accounting for 44 % of the year). The first half of this period was influenced by temperatures fluctuating between 10–15 °C while the second half was characterized by temperatures >15 °C.

**Fig. 4:**
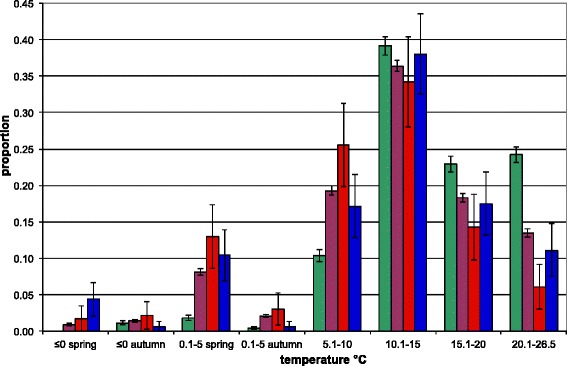
Proportions of the total numbers of each stage collected over the 6 year monitoring period according to temperature category. Proportions are expressed as cumulative frequencies (with 95 % confidence intervals) reflecting the number of monitoring visits within a given temperature category (see Additional file 1: Table S1)

To analyze temperature in relation to daily questing activity, numbers of ticks per monitoring visit were grouped according to near-ground temperature category (Additional file 1: Table S1). During the whole monitoring period, the difference between near-ground temperatures within one monitoring visit were never >5 °C. The most frequently recorded near-ground temperature category was 10.1–15.0 °C (30 % monitoring visits). Near-ground temperature categories ≤10 °C (0.1–5.0 °C and 5.1–10.0 °C) were recorded in 47 % visits, while temperatures ≥15.1 °C were measured in 23 % visits. The greatest proportion of all stages collected over the 6 year monitoring period was at 10.1–15.0 °C; 39 % of all active larvae were recorded in this temperature range together with 36 % nymphs, 34 % females, and 38 % males (Fig. 4). While the proportions at 10.1–15.0 °C were similar for each developmental stage, this was not the case in other temperature categories. For example, larvae showed a greater relative abundance at temperatures >15 °C compared with all other stages.

**Fig. 5:**
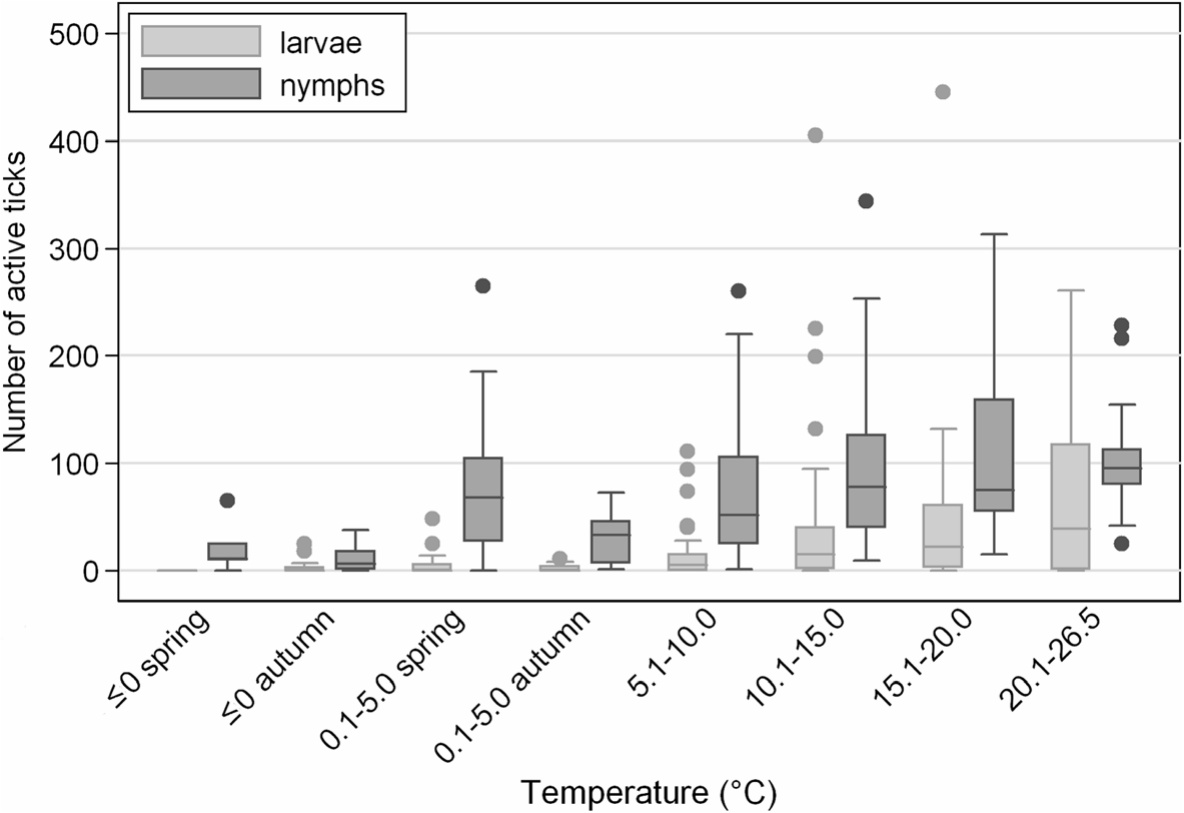
Box and whisker plot showing the numbers of larvae and nymphs collected over the 6 year monitoring period according to temperature category. Each plot shows the median, 25^th^ and 75^th^ percentiles, and range of questing tick numbers; outliers are shown as individual points and defined as >75^th^ percentile by ≥1.5 times the interquartile range (IQR) or <25^th^ percentile by ≥1.5 times the IQR. IQR is defined as 75^th^ percentile minus 25^th^ percentile. The observations represent individual days when ticks were collected. 50 % observations for the indicated temperature range lie within the box plot

The medians of questing nymphs and adults recorded at <10 °C were similar for spring and summer periods (*p* = 0.726). However, differences were revealed between spring and autumn periods at <10 °C (*p* = <0.001) and between summer and autumn periods at <10 °C (*p* = <0.001), with fewer questing ticks recorded in autumn (Additional file 1: Table S1 and Fig. 5). Larvae showed a comparatively low tolerance to low temperatures (Fig. 5).

### Questing activity of nymphs

**Fig. 6:**
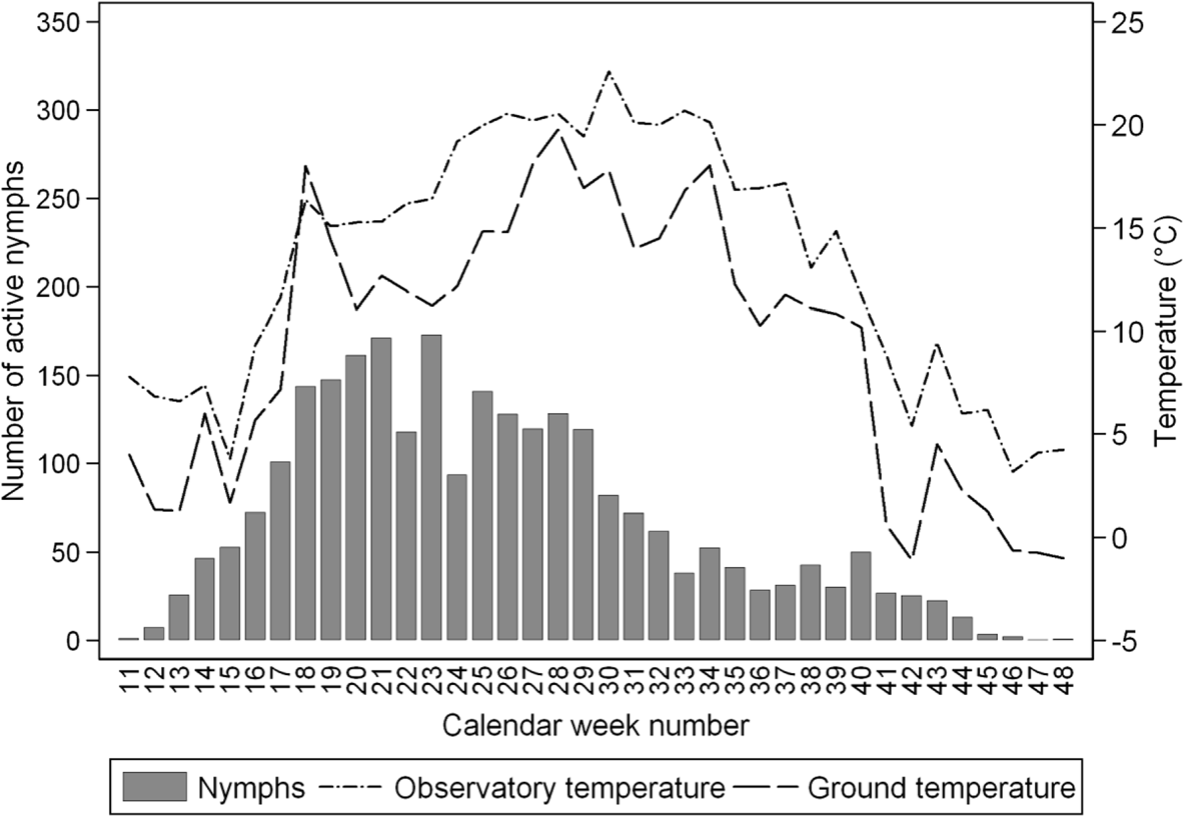
Time course of average numbers of host-seeking *Ixodes ricinus* nymphs compared to trends in the near-ground air temperatures and local meteorological temperatures. Temperatures (°C) were measured in the experimental plots and the respective standard temperature data were recorded by the Czech Hydrometeorological Institute (CHMI), Prague Libuš, during the monitoring period 2001–2006

Questing activity of nymphs was observed for 66.4 ± 3.6 % of the calendar year (Fig. 6). The mean onset of questing activity occurred at 12.4 ± 0.6 h of daylight and ended at 9.0 ± 0.4 h of daylight, at temperatures approaching freezing. Following the initiation of questing, numbers increased sharply to week 18 when both micro- and macroclimatic temperatures >10 °C. The sharp drop in mean temperature in week 15 was influenced by conditions in 2003, 2004, and 2005; although reflected in a decline in questing nymphs in those years, there was no effect on the overall mean number of questing nymphs. However, a sharp drop in number of questing nymphs around week 22 was recorded in all years, with a year-on-year shift of ≤2 weeks matched by a fall in near ground temperature to 10–12 °C. Numbers then recovered although not to peak levels, remaining so for July (weeks 25 to 29), and then declining during August (to week 36) (Fig. 6). A late peak in late September (week 40) coincided with the end of micro-and macroclimatic temperatures ≥10 °C. Temporary increases in temperature at the end of the questing period in 2001 and 2004 did not affect the 6 year mean decline in questing numbers.

**Fig. 7:**
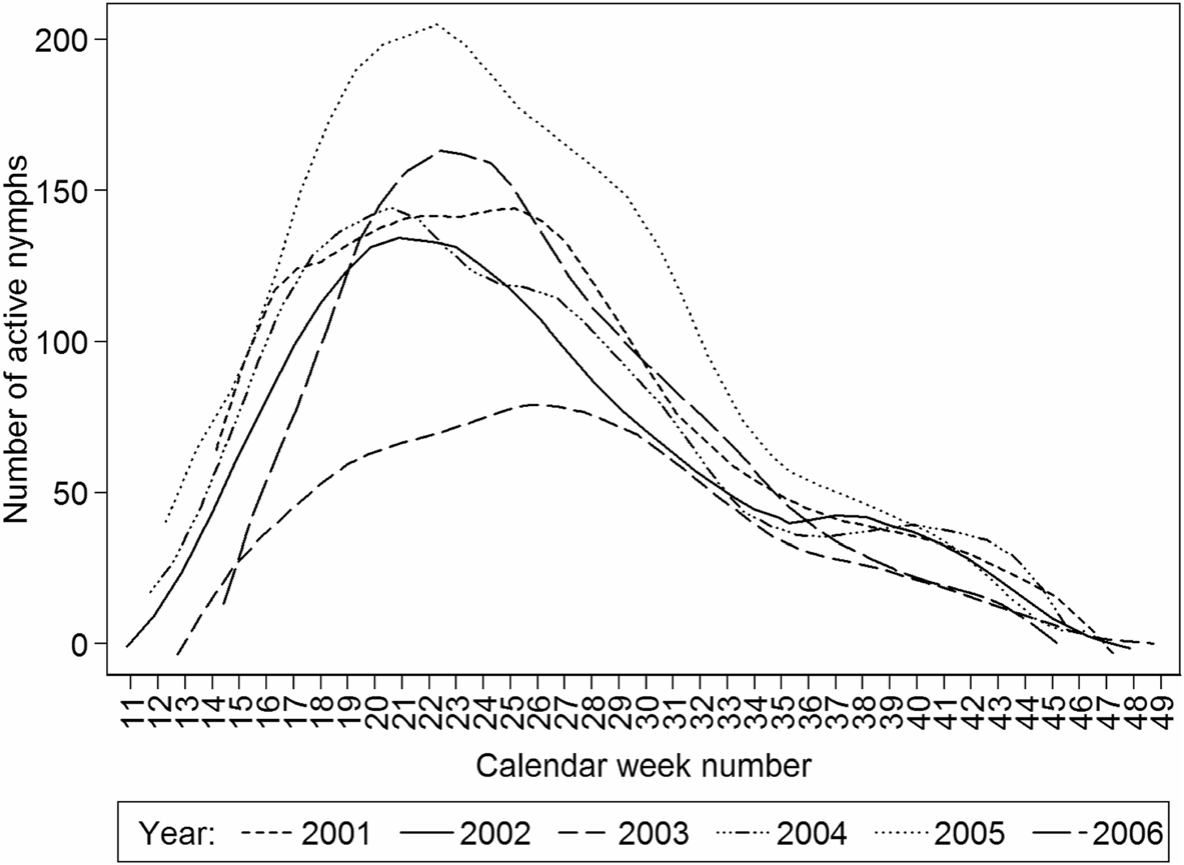
Comparison of the annual seasonal questing activity patterns of *Ixodes ricinus* nymphs. The data are smoothed by locally weighted regression (LOWESS)

**Fig. 8:**
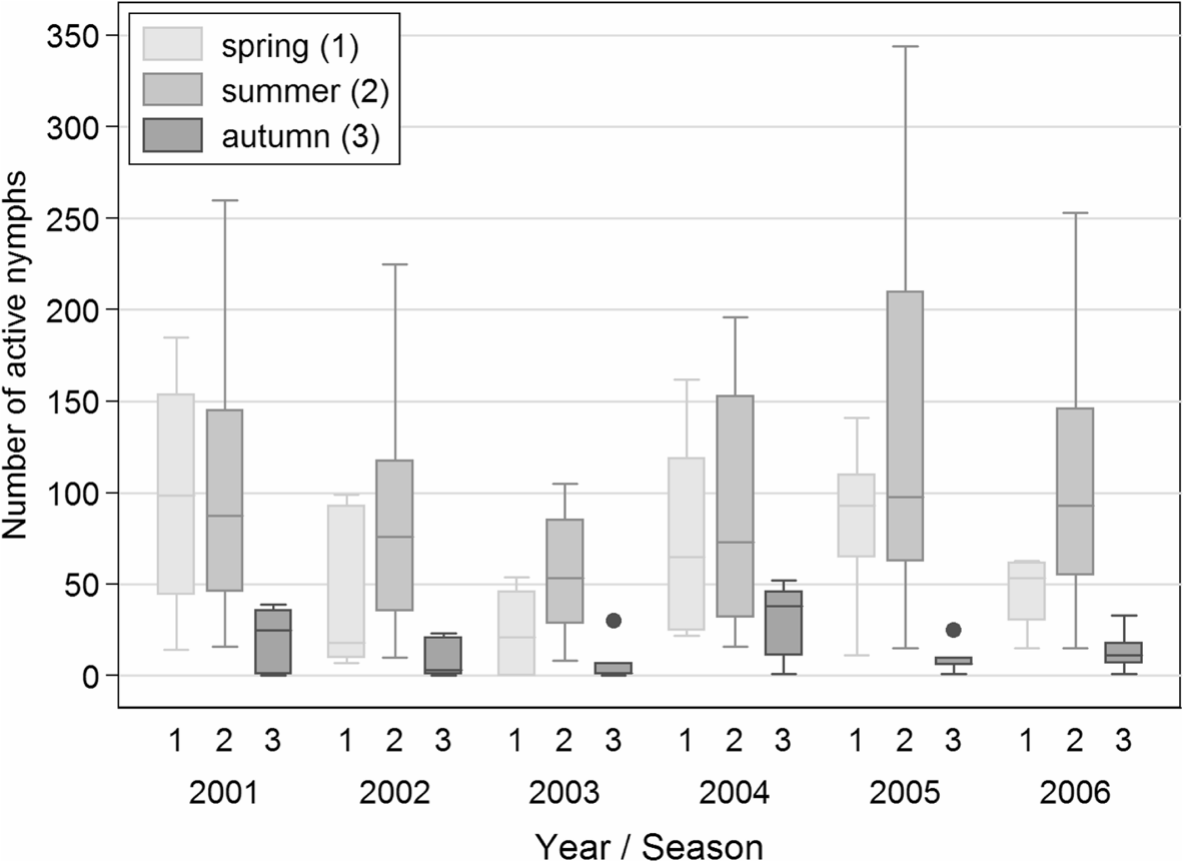
Comparison of the inter-annual seasonal questing activity of nymphs by temperature category. Box and whisker plot showing the median, quartiles, range and outliers of questing nymphs during different annual temperature phases of the monitoring period (see Fig. 5 legend for full description). See Methods for definition of spring (1), summer (2), and autumn (3)

Comparison of the numbers of questing nymphs by year using regression analysis (LOWESS) revealed similar annual seasonal activity curves, typically with a major spring peak and a minor autumn peak of questing activity (Fig. 7). The main peak is always slightly asymmetrical in shape. However, in 2005, both the numbers of nymphs (*p* < 0.001) and the shape of the curve (*p* = 0.005) differ significantly. Furthermore, the curve for 2003 shows a different pattern to all other years (*p* < 0.001) and comparatively low *I. ricinus* counts in summer months (*p* = 0.001). When the numbers of questing nymphs were compared during different temperature phases of each annual monitoring period, differences for 2003 and 2005 were most apparent at normal daily temperatures > 10 °C (Fig. 8 summer). Comparison of 2003 and 2005 with the rest showed that summer 2003 was significantly lower (*p* = 0.017) while summer 2005 had a tendency to increased numbers of nymphs (*p* = 0.053); numbers in spring and autumn of 2003 and 2005 were not significantly different from other years (*p* > 0.05).

Using the multiple Poisson regression model to compare nymphal activity with selected variables (Table 1), near-ground temperature (IRR = 1.009), length of daylight (IRR = 1.006), and RH (IRR = 1.004) were significant predictors of questing numbers (*p* < 0.001). Comparing years, with 2001 as the reference year because it was at the beginning of the monitoring period and is relatively typical (Fig. 7), the numbers of nymphs differed significantly between years (*p* = 0.007–< 0.001) except for 2004, which was not significantly different from 2001 (Table 1). When each year was compared with the grand mean, all years differed (*p* = 0.009–< 0.001) except for 2006 (*p* = 0.114) indicating that 2006 was an average year (data not shown). Similarly, comparing spring with summer and autumn seasons, numbers of nymphs differed significantly between seasons being lower in summer and autumn than in spring (*p* < 0.001). When adjusted for temperature and length of daylight, spring (as defined in Methods) is the dominant period for nymphal activity.

**Table 1:** Parameter estimates for the occurrence of questing *Ixodes ricinus* nymphs depending on selected predictors

Independent variable	IRR^1^	Standard error	95 % CI^2^	*p*-value
Near-ground temperature	1.009	0.002	1.005–1.012	<0.001
daylight length	1.006	0.000	1.006	<0.001
relative humidity (RH)	1.004	0.001	1.003–1.006	<0.001
year				<0.001
2002	0.821	0.023	0.777–0.868	<0.001
2003	0.539	0.018	0.506–0.574	<0.001
2004	0.949	0.027	0.898–1.003	0.062
2005	1.276	0.033	1.214–1.342	<0.001
2006	0.924	0.027	0.874–0.979	0.007
period				<0.001
summer	0.767	0.023	0.723–0.814	<0.001
autumn	0.785	0.044	0.703–0.876	<0.001

Parameter estimates were determined from the Poisson regression model. Temperature and daylight length are continuous variables whereas year and seasonal period are categorical variables. The reference year is taken as 2001 and the reference season as spring. IRR_temperature_ =1.009 indicates that if the temperature increases by 1 °C, the incidence increases 1.009 times (by 0.9 %). IRR_daylight_ =1.0062 indicates that for a change of 1 min, the incidence increases 1.0062 times (by 0.62 %). IRR_RH_ = 1.004 indicates that for a change of 1 % RH, the incidence increases 1.004 times (by 0.4 %). For year, IRR values less or greater than 1 indicate that the number of nymphs in the given year is lower or higher than in 2001, the reference year. For period, IRR values are less than 1 indicating that numbers of questing nymphs are 23 % (summer) and 44 % (autumn) less than during the spring period. All the independent variables were analysed simultaneously in one model so that their effects are adjusted for each other. All IRR values are significant (*p* = 0.007 or <0.001) except for 2004, which was not significantly different from 2001

*IRR* incidence rate ratio; *95 % CI* 95 % confidence interval

### Comparison of ecoclimate and meteorological observations

**Fig. 9:**
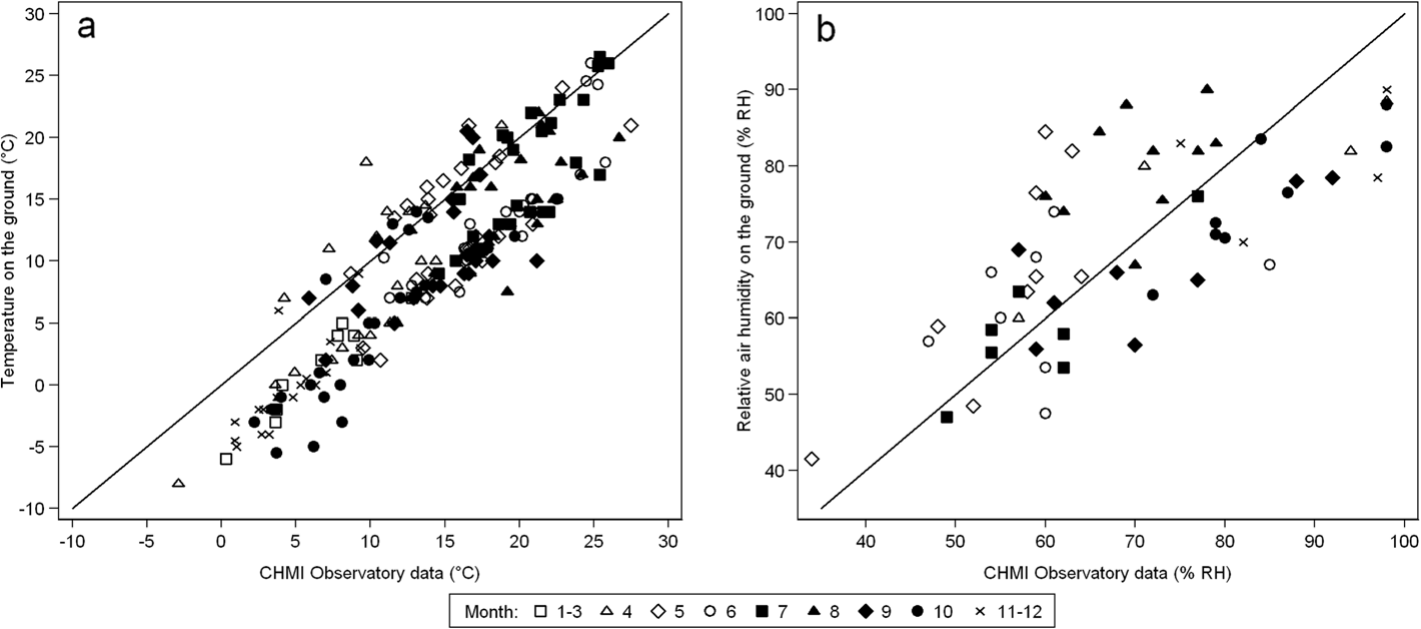
Comparison of the temperature (**a**) and relative humidity (**b**) measured in the monitoring plots with the coincident meteorological data recorded by the Czech Hydrometeorological Institute. Each data point represents the paired readings of ecoclimate with CHMI data measured on the day of monitoring for questing ticks

Near-ground air temperature and RH measured directly in the monitoring plots were compared with coincident meteorological data registered by the CHMI observatory at 10.00 h (Fig. 9). Not surprisingly, the temperature data are strongly dependent (*r*_*s*_ = 0.86); however, ~70 % recordings in the monitoring plots were 4–5 °C lower (with a maximum difference of 10 °C) than those recorded by CHMI, as shown by the distribution of temperature records around the diagonal representing full agreement in Fig. 9a. In fact, the comparative data fall into two clearly defined groups separated by a distinct gap with no observations. Analysis of individual seasons (2001–2006) reproduced this double grouping pattern indicating that it was not an artefact (data not shown). Similar temperature recordings, and slightly higher near-ground temperatures compared with CHMI records, fell mainly within the summer period, accounting for 82 % of the similar group of data (in comparison with 10 % in spring and 8 % in autumn). Near-ground temperature appears to be a better predictor of numbers of nymphs compared with CHMI temperature (*r*_*s*_ = 0.53 and 0.45, respectively) although local weather station temperature forecasts are a good proxy. Furthermore, the correlation between daylight length with CHMI temperature is stronger than with near-ground local temperature, and this complicates the differentiation of temperature and daylight length effects. Hence near-ground temperature, reflecting the conditions in the monitoring site, is the preferred predictor of nymphal activity. Near-ground RH measurements, recorded during 2005 and 2006, ranged from 50 to 95 % RH and, unlike temperatures, varied widely although RH still showed a strong correlation with CHMI recordings (*r*_*s*_ = 0.69). In spring, the RH data measured on the monitoring plots were mostly higher than recordings from the CHMI observatory, while the opposite was observed in autumn (Fig. 9b). Near-ground temperature was a better predictor of tick (nymph and adult) activity than RH (Table 1 and Additional file 2: Figure S1).

## Discussion

The primary aim of the 6-year study was to determine how well routine weather forecasts predict the risk to humans of being bitten by the most important disease vector in Europe, *I. ricinus.* However, the extensive dataset collected during the study (one of the largest ever reported for a major disease vector), enabled us to examine various aspects of tick questing dynamics, seasonality, and inter-annual variation in a well-defined tick population.

The questing larval population was under-represented as demonstrated by comparison with the number of nymphs recorded (5690 larvae compared with 15,405 nymphs). Most likely this was because the distribution of larvae is highly aggregated, larvae having hatched from an egg mass with minimal ensuing dispersal, although other factors such as host seeking success and height of questing in the vegetation, may have contributed. The influence of factors identified by others, such as vegetation height [3, 22, 23], was minimized by careful selection of the monitored plots (see Introduction and Methods). For both nymphs and adults, questing numbers may include ticks that were captured more than once because ticks were not removed; nevertheless, the numbers recorded represent the risk to humans receiving a tick bite. Ratios of nymphs to adults have been shown to vary greatly, depending on the state of the local biocenosis (especially the occurrence of suitable hosts for nymphs) and meteorological conditions [24, 25]. The overall ratio of adult males to females of 1:0.78 reflects the longer time spent by males on vegetation seeking repeat matings [26]. Overall, the relative differences in numbers of the different stages indicate the numbers of sampled nymphs are the most robust.

To account for the observed differences in sampling bias, numbers of active stages were standardized in order to compare their overall seasonal dynamics of questing activity (Fig. 3). The observations that female tick numbers peaked first and that the second peak of male numbers occurred in summer rather than autumn, have not been reported previously. Although all stages showed a bimodal activity curve, the second peak of activity was not clearly demarcated as there was a relatively high level of activity during the summer months. Previous reports have recorded bimodal and unimodal activity, varying according to location and habitat [3, 27, 28]. Consequently, there was a substantial risk of human contact with ticks throughout the 35 ± 2 weeks of tick activity (~70 % of the calendar year). This contact period corresponded to the duration of nymphal questing activity and an estimated 12.3 active nymphs per day per 100 m^2^. Although initiation and cessation of questing activity correlated with temperature, in autumn, nymphs and adult *I. ricinus* ticks were more sensitive to a temperature drop, entering behavioural diapause at near-ground temperatures greater than those associated with the onset of questing behaviour in the spring period. This difference can be explained by the influence of the increasing number of daylight hours in spring compared with the shortening daylight period in autumn. Thus in summer, short-term drops in temperature have a lower impact on questing behaviour than they do in autumn. The seemingly low tolerance of larvae to low temperatures (Fig. 5) may be due to the temperature trigger for hatching [3] (although the results contradict the observation that threshold temperatures for egg development are 5 °C and 7 °C for eggs deposited by autumn and spring-fed adult females, respectively [29]). Comparatively low temperature sensitivity is reflected in the seasonal dynamics of larval questing behavior (Fig. 3) and the co-occurrence of larvae with other stages of *I. ricinus* ticks (Fig. 2) [30].

The co-occurrence of larvae with nymphs is considered a risk factor for humans because of its modeled contribution to the intensity of TBE virus infection in tick populations as represented by the basic reproductive number of the infection, R_0_ (the average number of secondarily infected individuals arising from one primary-infected individual placed in an entirely susceptible population). The greater R_0_ for an infection, the greater the threat to humans and other susceptible animals [31]. For TBE virus, R0 ≅ 1 indicating the tenuous survival of the virus in nature, which is thought to rely heavily on co-feeding of infected nymphs with uninfected larvae [31, 32]. For TBE virus transmission to occur between immature stages of *I. ricinus*, infected and uninfected ticks must feed on the same individual host and at overlapping periods of time (so-called ‘co-feeding transmission’). However, comparison of the questing activity patterns of larvae and nymphs reveals considerable intra- and interannual variation (Figs. 2, 3 and 5; Additional file 1: Table S1). As there was no occasion when larvae were present and nymphs were absent, variation in coincident larval and nymphal questing activity was determined by larvae. Larvae tend to be overlooked as risk factors for TBE virus infection because infected larvae are rarely detected. Nevertheless, larvae can acquire infections through vertical transmission from the infected parent(s) to the egg, and (at least for TBE virus) amplify the infection levels through co-feeding of infected and uninfected larvae [33–35]. Considering the observed variations in coincident questing activity, and the different hosts on which larvae and nymphs typically feed [36–41], transmission between infected and uninfected larvae (intrastadial co-feeding transmission) should be considered in estimating R_0_ and in predicting the risk to humans of being bitten by infected nymphs, particularly for TBE virus.

Given the observed risk of human contact with ticks for ~70 % year, is it possible to predict the start and end of the ‘at risk’ period based on meterological conditions? The data indicate air temperature recorded at the local meterological station was a predictor of tick activity in that near ground temperature is strongly dependent on ambient temperature. Day length appeared to influence activity at the lowest temperatures (i.e. <5 °C); in spring relatively more activity was recorded compared with comparable temperatures in autumn. Hence temperature was a better predictor of the cessation of tick activity in autumn than the initiation of questing activity in spring, at least for nymphs and adults. However, although day length was shown to be a significant predictor of nymphal activity, this is strictly tied to specific geographical location. For example, in Algeria and the Crimea, ticks are active in winter months when day length is reduced and temperatures are similar to those during the spring–autumn period in Central Europe [27, 42]. Indeed, continuous activity extending throughout winter has been recorded in Central Europe (Berlin) during a notably mild winter [43].

Despite some ability to predict tick activity based on temperature, day length, and relative humidity, considerable variation was found between years during the 6 year study. This was particularly evident in 2003 and 2005, although the impact within each of these years was greatest during the summer period rather than at the initiation or cessation of activity. During summer, the similarity between near-ground temperatures and air temperature records at the weather station was greatest. In 2003, the spring and summer months were extremely dry (deficiency in precipitation totals compared with 30 year average: March, 40 %; April 47 %; June 48 %, August 40 %, September 60 %) accompanied by temperatures exceeding the 30 year average by 2.9 °C in May, 4.1 °C in June and 3.8 °C in August (Additional file 3: Figure S2). The atypical conditions of 2003 were preceded by an extreme event in 2002 in the form of major flooding in Central Europe [14]. Although 2003 coincided with the lowest recorded numbers of nymphs, this was not the case for other stages, and there was no obvious impact of flooding on tick numbers in 2002 although the monitoring site was not flooded. The depressed activity of nymphs during the meteorologically anomalous conditions of 2003, is an indication that changes in climate can impact the threat to humans posed by *I. ricinus*. For 2005, heightened summer and depressed autumn nymphal activity (Figs. 7 and 8) correlated with hot, humid summer and hot, dry autumn months (Additional file 3: Figure S2). Despite these meteorologically abnormal years, questing activity of nymphs in the following years (2004 and 2006) returned to typical levels.

Based on the evaluation of results for 2001–2003, the first version of a computer program (acronym TICKPRO) was prepared in cooperation with CHMI, which forecasts the level of *I. ricinus* questing activity within a 1–4 day horizon [44]. The program is based on the weather forecast routinely produced at the CHMI as well as mathematical models describing the correlation of meteorological factors with the questing activity of *I. ricinus*. During 2004–2006, the forecasts were tested and the program modified accordingly. Final adjustments to the program were undertaken during 2007 and 2008 when the forecasts were made public. Currently, 4-day tick activity predictions are produced twice weekly for the period from March to November with a resolution of ten levels of questing activity of *I. ricinus*, and hence the risk of tick bite during outdoor activities. The prediction is publically accessible on the websites of CHMI (www.chmi.cz/portal/dt?menu=JSPTabContainer/P9_0_Predpovedi/P9_1_Pocasi/P9_1_1_Cesko/P9_1_1_6_Klistata), National Institute of Public Health (www.szu.cz/tema/prevence/predpoved-aktivity-klistete-obecneho-na-uzemi-ceske-1), and the Ministry of Health of the Czech Republic (www.mzcr/verejne/dokumenty/predpoved-aktivity-klistat). It is accompanied by instructions on preventive measures at various levels of risk of tick attack. In high risk situations, it is broadcast on TV news, radio, and in newspapers. Detailed analysis presented in this manuscript will be used to expand the forecast to epidemiological significance: from the risk of tick attack to the risk of possible infection with TBE virus.

## Conclusions

Six years of replicated weekly monitoring of a carefully selected field site provided robust data for the comparison of seasonal questing activity of *I. ricinus* with abiotic conditions. Weather predictions (particularly for temperature) combined with daylight length, proved to be statistically good predictors of *I. ricinus* nymph questing activity, which presents the greatest risk to humans of acquiring tick-borne infections. Co-occurrence data for larvae and nymphs support the need to consider intrastadial rather than interstadial co-feeding pathogen transmission as being critical to the survival of tick-borne encephalitis virus in nature. Although extreme weather events appeared to impact questing populations, tick numbers recovered quickly in subsequent years. The approach described can be used to provide the basis for a weather-based prognosis of tick activity.

## Additional files

Additional file 1: Table S1. Overview of questing stages of *Ixodes ricinus* ticks recorded in different near-ground air temperature categories during the monitoring periods in 2001–2006. (DOCM 19 kb) Additional file 2: Figure S1. Questing activity of *Ixodes ricinus* (nymphs and adults) recorded at different near-ground temperatures and relative air humidities during 2005 and 2006. (DOCM 104 kb) Additional file 3: Figure S2. Annual climagrams for 2003 and 2005. x-axis: deviations of monthly average air temperatures in °C from 30-years average of monthly air temperatures; y-axis: deviations of monthly sums of precipitation in % from 30-years average of monthly sums of precipitation. Point of axes intersection represents 30-years monthly averages. (DOC 54 kb)
