# Supplementary material for: Abiotic predictors and annual seasonal dynamics of Ixodes ricinus, the major disease vector of Central Europe
Source: Parasit Vectors. 2015 Sep 18;8:478. doi: 10.1186/s13071-015-1092-y (PMC4575455; doi:10.1186/s13071-015-1092-y)
Supplement: Additional file 1: Table S1. — Overview of questing stages of Ixodes ricinus ticks recorded in different near-ground air temperature categories during the monitoring periods in 2001–2006. (DOCM 19 kb) [file 13071_2015_1092_MOESM1_ESM.docm]

**Table S1 Overview of questing stages of *Ixodes ricinus* ticks recorded in different near-ground air temperature categories during the monitoring periods in 2001-2006.**

| Temperature category (°C) | Number of visits | Registered from – to  (Month, Day) |  | Larvae | Nymphs | Males | Females |
| --- | --- | --- | --- | --- | --- | --- | --- |
| ≤ 0 | 7 | March 18 – April 18 | Total | 0 | 139 | 13 | 4 |
|  |  |  | Mean | 0 | 19.9 | 1.9 | 0.6 |
|  |  |  | Median | 0 | 11 | 0 | 0 |
| ≤ 0 | 22 | October 6 – December 1 | Total | 64 | 218 | 2 | 5 |
|  |  |  | Mean | 2.9 | 9.9 | 0.1 | 0.2 |
|  |  |  | Median | 0 | 6.5 | 0 | 0 |
| 0.1 – 5 | 17 | March 11 – May 24 | Total | 103 | 1248 | 31 | 30 |
|  |  |  | Mean | 6.4 | 78.0 | 1.9 | 1.9 |
|  |  |  | Median | 0 | 68 | 1.5 | 1 |
| 0.1 – 5 | 11 | September 11 – November 18 | Total | 24 | 324 | 2 | 7 |
|  |  |  | Mean | 2.2 | 29.5 | 0.2 | 0.6 |
|  |  |  | Median | 0 | 33 | 0 | 0 |
| 5.1 – 10 | 41 | March 16 – November 14 | Total | 589 | 2974 | 51 | 59 |
|  |  |  | Mean | 14.4 | 72.5 | 1.2 | 1.4 |
|  |  |  | Median | 5 | 52 | 1 | 1 |
| 10.1 – 15 | 63 | April 10 – October 25 | Total | 2227 | 5607 | 113 | 79 |
|  |  |  | Mean | 35.3 | 89.0 | 1.8 | 1.3 |
|  |  |  | Median | 15 | 78 | 1 | 1 |
| 15.1 – 20 | 27 | April 3 – September 25 | Total | 1304 | 2824 | 52 | 33 |
|  |  |  | Mean | 48.3 | 104.6 | 1.9 | 1.2 |
|  |  |  | Median | 22 | 75 | 2 | 1 |
| 20.1 – 26.5 | 20 | April 30 – September 11 | Total | 1379 | 2071 | 33 | 14 |
|  |  |  | Mean | 69.0 | 103.6 | 1.7 | 0.7 |
|  |  |  | Median | 39 | 95.5 | 1 | 1 |
