# Supplementary material for: Abiotic predictors and annual seasonal dynamics of Ixodes ricinus, the major disease vector of Central Europe
Source: Parasit Vectors. 2015 Sep 18;8:478. doi: 10.1186/s13071-015-1092-y (PMC4575455; doi:10.1186/s13071-015-1092-y)
Supplement: Additional file 2: Figure S1. — Questing activity of Ixodes ricinus (nymphs and adults) recorded at different near-ground temperatures and relative air humidities during 2005 and 2006. (DOCM 104 kb) [file 13071_2015_1092_MOESM2_ESM.docm]

**Figure S1 Questing activity of *I. ricinus* (nymphs and adults) recorded at different near-ground temperatures and relative air humidities during 2005 and 2006.**
