# Supplementary material for: Abiotic predictors and annual seasonal dynamics of Ixodes ricinus, the major disease vector of Central Europe
Source: Parasit Vectors. 2015 Sep 18;8:478. doi: 10.1186/s13071-015-1092-y (PMC4575455; doi:10.1186/s13071-015-1092-y)
Supplement: Additional file 3: Figure S2. — Annual climagrams for 2003 and 2005. x-axis: deviations of monthly average air temperatures in °C from 30-years average of monthly air temperatures; y-axis: deviations of monthly sums of precipitation in % from 30-years average of monthly sums of precipitation. Point of axes intersection represents 30-years monthly averages. (DOC 54 kb) [file 13071_2015_1092_MOESM3_ESM.doc]

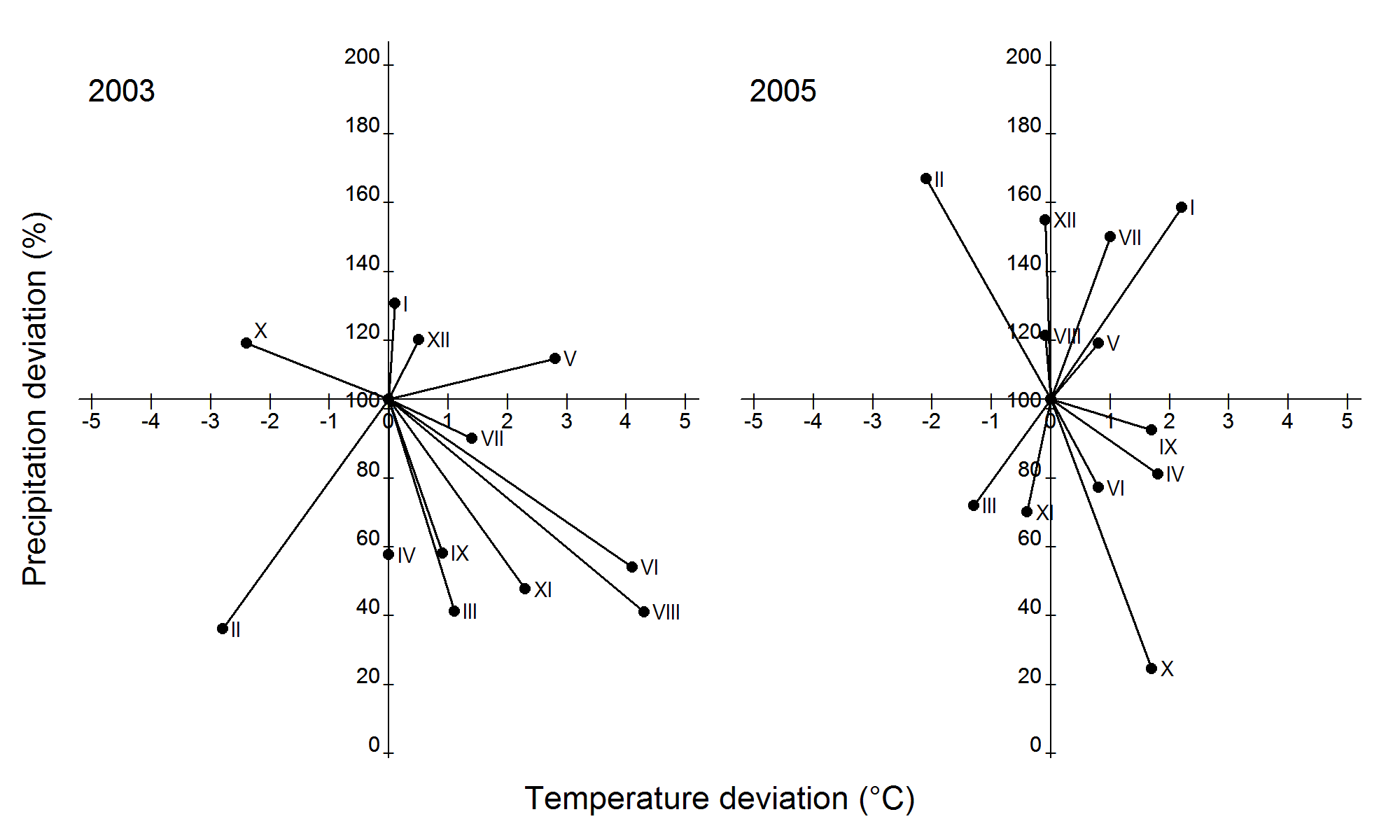


**Figure S2 Annual climagram 2003 and 2005.** x-axis: deviations of monthly average air temperatures in °C (dt) from 30-years average of monthly air temperatures; y-axis: deviations of monthly sums of precipitation in % (% R) from 30-years average of monthly sums of precipitation. Point of axes intersection represents 30-years monthly averages.
